# Infectious Complications in Head and Neck Cancer Patients Treated with Cetuximab: Propensity Score and Instrumental Variable Analysis

**DOI:** 10.1371/journal.pone.0050163

**Published:** 2012-11-28

**Authors:** Ching-Chih Lee, Hsu-Chueh Ho, Shih-Hsuan Hsiao, Tza-Ta Huang, Hon-Yi Lin, Szu-Chin Li, Pesus Chou, Yu-Chieh Su

**Affiliations:** 1 Department of Otolaryngology, Buddhist Dalin Tzu Chi General Hospital, Chiayi, Taiwan; 2 Community Medicine Research Center and Institute of Public Health, National Yang-Ming University, Taipei, Taiwan; 3 Cancer Center, Buddhist Dalin Tzu Chi General Hospital, Chiayi, Taiwan; 4 Division of Hematology-Oncology, Department of Internal Medicine, Buddhist Dalin Tzu Chi General Hospital, Chiayi, Taiwan; 5 Department of Radiation Oncology, Buddhist Dalin Tzu Chi General Hospital, Chiayi, Taiwan; 6 School of Medicine, Tzu Chi University, Hualien, Taiwan; 7 Department of Oral and Maxillofacial Surgery, Changhua Chritian Hospital, You-Lin Branch, You-Lin, Taiwan; Technische Universitaet Muenchen, Germany

## Abstract

**Background:**

To compare the infection rates between cetuximab-treated patients with head and neck cancers (HNC) and untreated patients.

**Methodology:**

A national cohort of 1083 HNC patients identified in 2010 from the Taiwan National Health Insurance Research Database was established. After patients were followed for one year, propensity score analysis and instrumental variable analysis were performed to assess the association between cetuximab therapy and the infection rates.

**Results:**

HNC patients receiving cetuximab (n = 158) were older, had lower SES, and resided more frequently in rural areas as compared to those without cetuximab therapy. 125 patients, 32 (20.3%) in the group using cetuximab and 93 (10.1%) in the group not using it presented infections. The propensity score analysis revealed a 2.3-fold (adjusted odds ratio [OR] = 2.27; 95% CI, 1.46–3.54; P = 0.001) increased risk for infection in HNC patients treated with cetuximab. However, using IVA, the average treatment effect of cetuximab was not statistically associated with increased risk of infection (OR, 0.87; 95% CI, 0.61–1.14).

**Conclusions:**

Cetuximab therapy was not statistically associated with infection rate in HNC patients. However, older HNC patients using cetuximab may incur up to 33% infection rate during one year. Particular attention should be given to older HNC patients treated with cetuximab.

## Introduction

The epidermal growth factor receptor (EGFR)-targeting IgG1 monoclonal antibody, cetuximab, is a breakthrough in targeted therapy for head and neck cancers, especially among patients with recurrent or metastatic disease [1]. In patients with locally advanced head and neck cancer, radiotherapy in combination with cetuximab has prolonged the median overall survival in a statistically significant manner when compared to radiotherapy alone [2]. In head and neck cancer patients with recurrent or metastatic squamous cell carcinoma, cetuximab in combination with platinum-fluorouracil chemotherapy improved overall survival when given as first-line treatment [3]. Recently, cisplatin-based chemoradiation in combination with cetuximab led to a complete response rate of 71% among participants in a phase II study that enrolled advanced head and neck cancer patients [4].

**Table 1:** Demographic characteristics for head and cancer patients by treatment modality (n = 1083).

Variables	With cetuximab(n = 158)	Without cetuximab (n = 925)	P-value
	n(%)	n(%)	
Age, yr			<0.001
Mean±SD	67±13	55±10	
Gender			0.363
Male	150(95)	892(96)	
Female	8(5)	33(4)	
Charlson Comorbidity Index Score			0.237
= 0	89(56)	474(51)	
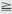 1	69(44)	451(49)	
Socioeconomic status			0.007
High ( 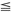 NT$20001 or US$626 )	25(16)	239(26)	
Low ( 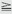 NT$20000 or US$625 )	133(84)	686(74)	
Urbanization level			0.035
Urban/Suburban	97(61)	646(70)	
Rural	61(39)	279(30)	
Region of residence			0.041
Northern/Central	112(71)	724(78)	
Southern/Eastern	46(29)	201(22)	
Treatment modality			0.302
Surgery+Chemotherapy+Radiotherapy	76(48)	486(53)	
Chemotherapy/Chemotherapy+Radiotherapy	82(52)	439(47)	

Previous studies reported that the administration of cetuximab does not alter or compromise the delivery of scheduled radiation doses or the pharmacokinetics of chemotherapy [1]. They also concluded that adverse side effects, such as skin reactions, are tolerable, and adverse pulmonary events are not statistically more frequent in patients receiving cetuximab [5], [6]. However, several series revealed an increased risk of infection events, neutropenia, or pulmonary adverse reactions, in patients treated with cetuximab. In a meta-analysis, patients treated with cetuximab incurred an additional 12% risk for developing severe neutropenia [7]. A higher rate of high-grade infections was observed with the use of cetuximab in addition to chemotherapy in a randomized phase III study [8]. Increased dyspnea and respiratory insufficiency were noted in head and neck cancer patients undergoing cetuximab therapy [9]. Death due to pneumonia was observed in patients with locoregionally advanced head and neck cancer who were administered a concurrent cetuximab, cisplatin, and boost radiotherapy regimen that was not recommended outside of the clinical trial setting [10].

**Figure 1:**
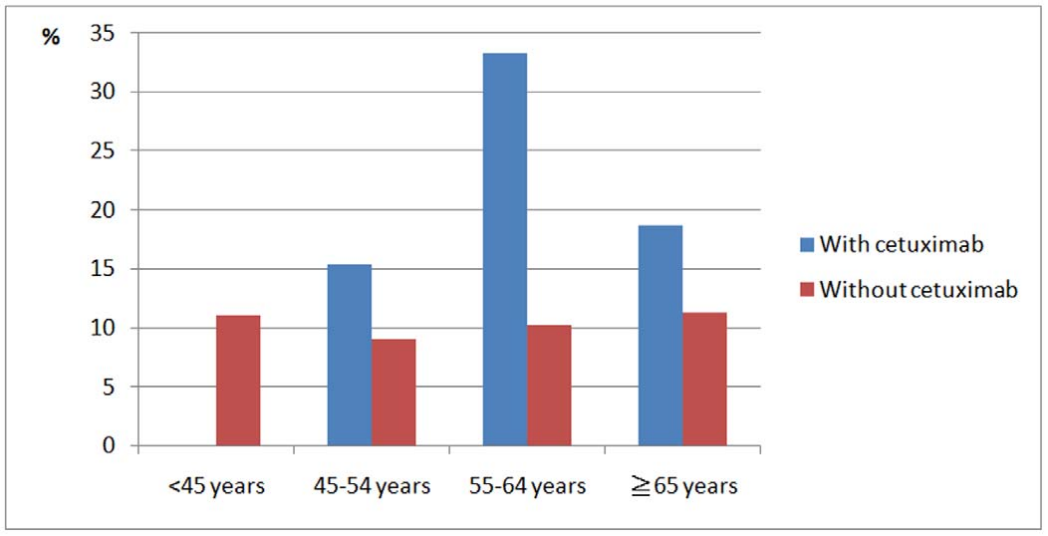
Infectious complications in head and neck cancer patients.

The purpose of this study was to examine the incidence of infection events in head and neck cancer patients identified through the National Health Insurance Research Database (NHIRD) in Taiwan. This allowed for a comparison of the risk of infection events between head and neck cancer patients receiving cetuximab therapy and those who were not treated with this compound. It also provided an opportunity to outline follow-up suggestions for cetuximab-treated head and neck cancer patients. Propensity score analysis and instrumental variable analysis techniques were utilized to minimize the selection bias in observational medical studies, such as our NHIRD [11], [12].

## Materials and Methods

### Ethics Statement

This study was initiated after approval by the Institutional Review Board of the Buddhist Dalin Tzu Chi General Hospital, Taiwan (IRB B10001018). Since all identifying personal information was stripped from the secondary files before analysis, the review board waived the requirement for written informed consent from the patients involved.

### NHIRD Dataset

Since 1995, the National Health Insurance program in Taiwan has enrolled up to 99% of the Taiwanese population and is contracted with 97% of the medical providers [13]. This study utilized the 2010 NHIRD published by Taiwan’s National Health Research Institutes. The NHIRD includes all prescribed medication and chemotherapy regimens. Information on tobacco use, dietary habits, and body mass index were not included in this database.

**Figure 2:**
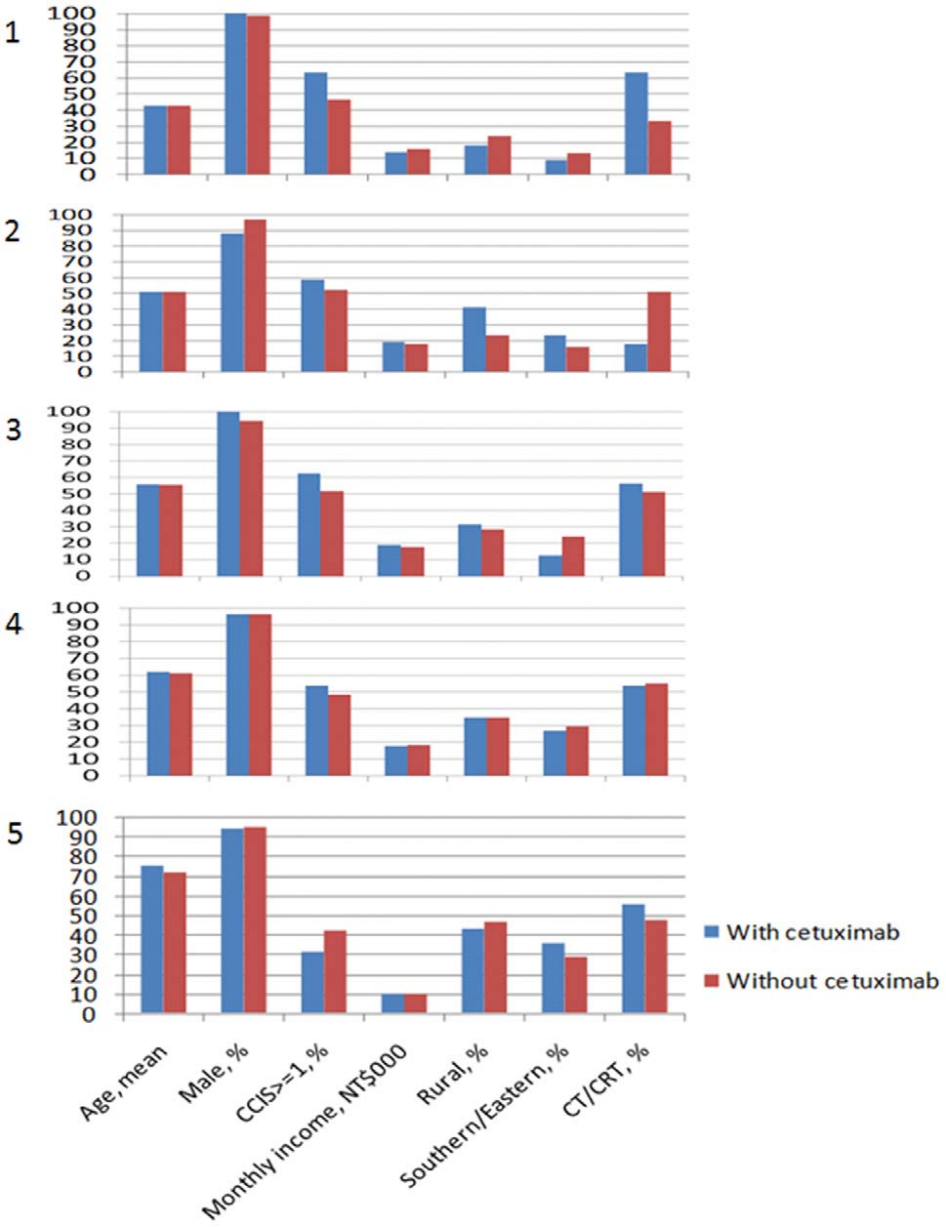
Distribution of explanatory variables between patients receiving cetuximab and those not receiving cetuximab for propensity score quintiles ranging from 1 (least likely to receive cetuximab) to 5 (most likely to receive cetxuimab).

### Study Sample Inclusion and Exclusion Criteria

According to the NHI treatment guidelines in Taiwan, cetuximab was approved for use in oropharyngeal, hypopharyngeal, and laryngeal cancer in patients who underwent radiotherapy and meeting any of the following criteria: 1) age 70 or more, 2) impaired renal function with creatinine clearance rates less than 50 ml/min, 3) hearing impairment with average pure tone audiometry over 25 dB, or 4) intolerance to platinum-based chemotherapy. The study population consisted of patients with head and neck cancer (identified according to the *International Classification of Diseases, Ninth Revision, Clinical Modification* [ICD-9-CM] codes including oropharyngeal cancer [146], hypopharyngeal cancer [148], and laryngeal cancer [161]) who were over 20 years of age and underwent radiotherapy, chemotherapy, or chemo-radiotherapy, with or without surgery, in 2010. A sample of 1083 patients was used based on the registry of catastrophic illness patient database and clinical exclusion criteria.

**Table 2:** Infection events in study population (n = 1083).

Mention term	With cetuximab (n = 158)	Without cetuximab (n = 925)	P-value
	n%	n%	
			<0.001
1.Laryngitis	1(0.6)	0	
3.Pneumonia	19(12)	39(4.2)	
4.Bronchitis	3(1.9)	9(1.0)	
5.Fever	0	4(0.4)	
6.Urinary tract infection	1(0.6)	0	
8.Viral infection	0	1(0.1)	
11.Herpes zoster	2(1.3)	0	
12.Septicemia	3(1.9)	14(1.5)	
15.Diverticulitis	1(0.6)	0	
17.Otitis media	0	1(0.1)	
18.Tonsillitis	0	3(0.3)	
19.Pharyngitis,epiglottitis, laryngopharyngitis	0	1(0.1)	
20.Tuberculosis	0	1(0.1)	
28.Pancreatitis	0	1(0.1)	
30.Acute upper respiratory infections	0	1(0.1)	
33.Cellulitis	1(0.6)	11(1.2)	
35.Infectious disease and parasitic disease	0	3(0.4)	
36.Others	1(0.6)	4(0.4)	
Infection (total = 125)	32(20.3)	93(10.1)	

### Measurements

A total of 1083 patients who met the inclusion and exclusion criteria were identified. Each patient was tracked from his or her index ambulatory visit in 2010 to identify outcomes including any type of infectious diseases. To maximize case ascertainment, only patients hospitalized for infection events were included. These patients were then linked to the administrative data to calculate the rate of infection events.

**Table 3:** Infection rate head and neck cancer patients treated with different treatment modality.

Variables	Infection event	Infection rate	P-value*
**With cetuximab** (n = 158)			0.581
Surgery+Chemotherapy+Radiotherapy (n = 76)	14	18.4	
Chemotherapy/Chemotherapy+Radiotherapy (n = 82)	18	22.0	
**Without cetuximab** (n = 925)			0.261
Surgery+Chemotherapy+Radiotherapy(n = 486)	54	11.1	
Chemotherapy/Chemotherapy+Radiotherapy(n = 439)	39	8.9	

* P value of Pearson’s chi-square test between the cetuximab group v.s without cetuximab group is <0.001.

We compared the outcomes for patients who underwent cetuximab therapy (the cetuximab group): chemotherapy (cisplatin/carboplatin-based), chemoradiotherapy, and surgery with chemoradiotherapy, and for those who did not receive cetuximab therapy (the non-cetuximab group): chemotherapy (cisplatin/carboplatin -based), chemoradiotherapy, and surgery with chemoradiotherapy. The two major groups (cetuximab versus non-cetuximab) were analyzed to explore the possible differences between cetuximab administration and infection events.

Patients were characterized by age, gender, treatment modality, comorbidities, individual socioeconomic status, and tumor site. In each patient, the comorbidities were based on the modified Charlson comorbidity index score, which was widely used in recent years for risk adjustment in administrative claims data sets [14]. The insurance amount from the database was used as a proxy for the individual socioeconomic status. The monthly income was classified into one of three categories: 1) low SES (less than NT$20000 or US$625 per month), and 2) high SES (NT$20001 or US$626 per month or more) [15].

The urbanization level of residence is also associated with cancer outcomes and was therefore included in our analysis [16]. We recorded the level of urbanization as urban and sub-urban (urbanization level 1–3) or rural (urbanization level 4–7).

**Table 4:** One-year cumulative risk of infection among the patients with cetuximab and those without (n = 1083)^a^.

Stratum	With cetuximab (n = 158)			Without cetuximab (n = 925)			P-value
	No.	% of stratum	Risk (%)	No.	% of stratum	Risk (%)	
1	12	5.6	0	204	94.4	9.3	0.268
2	15	6.9	20	202	93.1	10.9	0.286
3	16	7.4	31.3	201	92.6	9.5	0.007
4	25	11.5	24	192	88.5	9.9	0.038
5	90	41.7	20	126	58.3	11.1	0.07
Total	158		19.1	925		10.1	<0.001
							0.001^b^

a Stratum 1 had the strongest propensity for not receiving cetuximab therapy; stratum 5, for receiving cetuximab therapy.

b Cochran-Mantel-Haenszel statistics; adjusted odds ratio = 2.27, 95% confidence interval = 1.46–3.54.

### Statistical Analysis

The SAS (version 9.2; SAS Institute, Inc., Cary, NC, USA) and SPSS (version 15, SPSS Inc., Chicago, IL, USA) statistical packages were used to analyze the data. Pearson’s chi-square tests were used to explore the differences between categorical variables in the different treatment groups. Continuous variables were analyzed with one-way ANOVA. Multivariate analysis was conducted with propensity score analysis and instrumental variable analysis.

**Figure 3:**
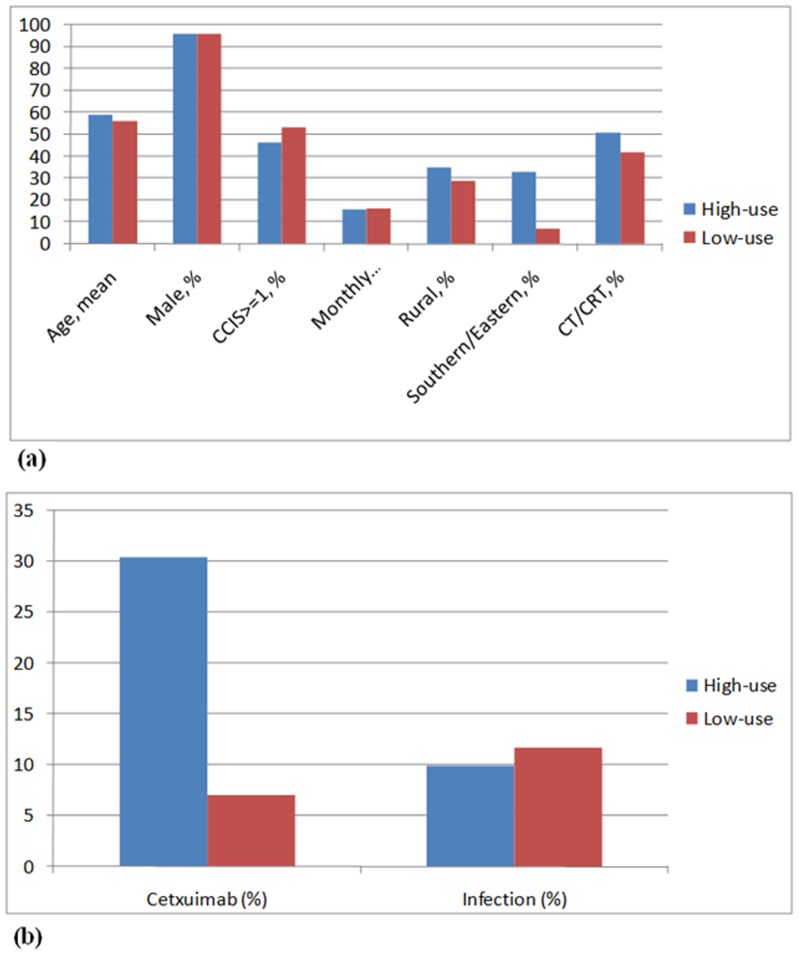
Distribution of explanatory variables between patients in high-use and low-use cetuximab hospitals (a) and infection rates (b).

#### (1) Propensity score

Propensity score stratification was applied to replace the wide host of confounding factors that may be present in an observational study with a variable of these factors [17], [18], [19]. To derive the propensity score in this study, patient characteristics were entered into a logistic regression model predicting selection for cetuximab therapy. The characteristics included age, gender, the Charlson Comorbidity Index score, urbanization and geographic area of residence, and treatment modality. The effect of cetuximab on the one-year infection rate was analyzed within each quintile. The Mantel-Haenszel odds ratio was calculated, in addition to performing the Cochran-Mantel-Haenszel χ^2^ test.

**Table 5:** Characteristics of head and neck cancer patients in high-cetuximab and low-cetuximab use hospitals (n = 611).

	High-use (n = 313)	Low-use (n = 298)	P-value
	n(%)	n(%)	
Age,yr (Mean±SD)	59±12	56±11	0.002
Male gender	302(96)	285(96)	0.590
Charlson Comorbidity Index Score			0.082
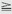 1	145(46)	159(53)	
Socioeconomic status			0.840
High (NT$20001 or 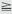 US$626)	81(26)	75(25)	
Urbanization level			0.094
Rural	109(35)	85(29)	
Region of residence			<0.001
Southern/Eastern	104(33)	21(7)	
Treatment			0.028
Chemotherapy/Chemotherapy+Radiotherapy	158(51)	124(42)	

* Parenthesis is percentage of patients in high-use or low-use hospitals.

#### (2) Instrumental variable analysis

Instrumental variable analysis from the Rubin Causal Model was used to account for both the measured and unmeasured confounding factors [20]. The instrumental variable was constructed by first calculating the proportion of head and neck cancer patients who received cetuximab in each hospital. Hospitals with one or more cases were included. The algorithm produced 36 hospitals. High- and low-use hospitals corresponded to the top and bottom tertiles of cetuximab utilization and were used as the binary instrumental variable for the binary treatment assignment. An instrumental variable must be associated with outcomes through its correlation with treatment status (cetuximab) and not through other covariates. The instrumental variable estimate was calculated by the formula:



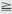
where “Hi” indicates a hospital with a high rate of cetuximab therapy administration and “Lo” indicates a hospital with a low rate of cetuximab therapy use.

**Table 6:** Marginal effect of cetuximab on infection event using instrumental variable analysis for one-year follow-up (n = 611).

	Odds ratio	(95%CI)	P-value
Cetuximab	0.87	(0.61–1.14)	0.319
Age, yrs	1.00	(0.95–1.21)	0.052
Male	1.08	(1.00–1.01)	0.248
Charlson ComorbidityIndex Score			
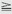 1	1.02	(0.97–1.08)	0.368
Socioeconomic status			
High ( 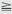 NT$20001 or US$626 )	0.95	(0.89–1.01)	0.076
Urbanization level			
Rural	0.99	(0.93–1.04)	0.614
Region			
Southern/Eastern	1.02	(0.95–1.09)	0.604
Treatment			
Chemotherapy/Chemotherapy+Radiotherapy	0.99	(0.94–1.04)	0.755

Abbreviation: 95% CI, 95% confidence interval.

We verified this assumption by comparing the baseline characteristics, including age at diagnosis, gender, the Charlson Comorbidity Index Score, and monthly income. The two-stage least squares method was used to estimate the effect of cetuximab by using instrumental variables.

## Results

In 1083 head and neck cancer patients, the median duration of follow-up was 6.5 months (interquartile range, 3.7–9 months). The mean age of the entire cohort was 57 years (standard deviation, 11 years). Among the participants, 96% were men and all patients were Asian. Among the patients with head and neck cancer, 158 were treated with cetuximab. Patients treated with cetuximab were older, and were more likely to have a lower socioeconomic status and to live in rural area, as compared to those who did not receive cetuximab therapy (Table 1).

At the end of the follow-up period, 125 patients had infection events, and of these, 32 (20.3%) were in the group using cetuximab and 93 (10.1%) were in the group that did not use it (Figure 1). HNC patients with cetuximab therapy aged 55–64 years incurred the highest infection rate of 33%.Table 2 shows the types of infection events for the two groups. Pneumonia was the most common infectious disease complication in both groups. In subgroup analysis, there was no statistical difference between the infection rate and treatment modality (surgery with adjuvant therapy versus chemotherapy or chemoradiotherpy) in cetuximab group or without cetuximab group (P = 0.581 and 0.261, respectively) (Table 3). Patients using cetuximab had an increased risk of infection events (P<0.001). Table 4 shows the infection rates for patients in each of the two groups after propensity score stratification. In most situations, patients with cetuximab therapy had higher infection rates. Figure 2 shows that most of the prognostic characteristics were well balanced within each propensity quintile. The P-value for Cochran-Mantel-Haenszel statistics comparing infection rates in patients receiving cetuximab therapy with infection rates among those not receiving cetuximab therapy, controlling for propensity scores, was 0.001. Patients treated with cetuximab had higher infection rates. The adjusted infection rates for patients treated with cetuximab were higher than for patients without cetuximab therapy (20.3% vs 10.1%; adjusted odds ratio [OR] = 2.27; 95% CI, 1.46–3.54; P = 0.001).

Propensity score analysis is unable to adjust for unmeasured confounders and selection biases, such as higher-risk patients who may be preferentially selected for cetuximab, thus producing apparently adverse outcomes for these groups. Among the IVA, most of the patients’ characteristics in high- and low-use cetuximab hospitals were well-balanced, similarly to the distribution of factors that one might hope for in a randomized trial (Figure 3a and Table 5). Cetuximab utilization varied widely across the different health care providers (3–90%). 87 patients had infection events, 14 (9.6%) in the high-use cetuximab hospitals and 73 (12.3%) in the low-use cetuximab hospitals (Figure 3b). By using IVA and the two-stage least squares analysis, we showed that cetuximab use was not statistically associated with infection events (OR, 0.87; 95% CI, 0.61–1.41; p = 0.319) (Table 6).

## Discussion

Limited data exist regarding whether cetuximab increases the rate of infections in patients with head and neck cancer. Most of the little information derived from randomized-controlled trials that were not designed to compare the infection rates among different treatment modalities, and many patients were often excluded from clinical trials. Data from day-by-day medical practices in the real world may reflect the true information. In propensity score analysis with adjusting observable confounding factors, the likelihood of developing infection events among head and neck cancer patients treated with cetuximab was 2.3-fold higher than among patients not receiving cetuximab therapy. Using IVA with adjusting measured and unmeasured confounding factors, the average treatment effect of cetuximab was not statistically associated with an increased risk of infection events in head and neck cancer patients.

The strengths of our analysis are the fact that it is a population-based study (n = 1083) in Taiwan, the nearly complete follow-up of any infectious events among the whole study population, and the regular monitoring of diagnosis accuracy and treatment by the National Health Insurance Bureau of Taiwan. Compared with randomized-controlled series or meta-analyses, the NHIRD is a real medical practice record that reflects the day-by-day medical care. Our series used two statistical methods, propensity score analysis and instrumental variable analysis. The propensity scores were used to stratify patients into five groups with similar propensity scores in order to reduce the effects of selection bias between the different treatment groups [18], [19], [21]. HNC patients treated with cetuximab were found to have increased rates of infection. Using IVA to control both the measured and unmeasured confounding factors, we did not find statistically differences between cetuximab and the rate of infections. The severity of comorbidities, the cancer stage, certain social factors such as employment, and patient preferences were difficult to capture correctly from the dataset. Referral selection may depend on the interactions between the comorbidities and cancer stage. All these unmeasured factors could produce significant bias using traditional approaches. Despite the efforts to simulate the randomization situation, propensity scores only adjusted for observable confounding variables. These observations imply that significant unaccounted residual bias exists among the propensity score methods and that IVA may be superior. The instrumental variable analysis was performed by comparing the baseline characteristics, and found that these factors were similar between the high- and low-use cetuximab institutions. The instrumental variable analysis produced less biased estimates.

There are few data evaluating the association between the infection rate and cetuximab therapy in patients with head and neck cancer. Increased risk for dyspnea and respiratory insufficiency had been reported in head and neck cancer patients treated with cetuximab [22]. Bonner et al. reported a 1.9% increase in the infection rate among HNC patients treated with radiotherapy and cetuximab, as compared to those treated with radiotherapy alone, and Burtness et al. revealed a 5% increase in infection rates in HNC patients treated with cetuximab and cisplatin, as compared to those treated with cisplatin alone [8], [23]. A recent meta-analysis found an additional 12% risk for advanced cancer patients treated with cetuximab and concurrent chemotherapy [24]. In the subgroup analysis, higher risk was observed in colorectal cancer patients (relative risk [RR] = 1.17; 95% CI, 1.04–1.32). This suggested that there are several plausible mechanisms to explain the increased rate of infectious complications in advanced cancer patients treated with cetuximab. EGF and EGF-like protein families, such as heparin-binding EGF-like growth factor (HB-EGF), are essential for cell proliferation, differentiation, and wound healing [25], [26]. Cetuximab may target the bone marrow EGF receptors, which are expressed on he surface of neutrophils and play key roles in their proliferation and differentiation. The suppressed bone marrow may further lead to neutropenia and increase the risk of infection. EGF could enhance reactive oxygen intermediates and IL-8 production by TNF-α-primed neutrophils [27]. This process could be suppressed by EGF receptor-selective tyrosine kinase inhibitors. However, subgroup analysis revealed that cetuximab was not associated with neutropenia in head and neck cancer patients receiving concurrent chemotherapy (RR = 1.22; 95% CI, 0.92–1.62). Besides the propensity score analysis, we tried to simulate a randomized study and balanced both the measured and the unmeasured characteristics in the different treatment groups with IVA. Using IVA and the two-stage least squares analysis, our series revealed that cetuximab was not associated, in a statistically significant way, with infection events.

Randomized-controlled trials cannot be undertaken in all situations where evidence is needed to provide treatment guidelines. Observational studies with adequate statistical analysis that have least bias are necessary to evaluate population effectiveness. Post-marketing surveillance is an important issue that could provide physicians, patients, and pharmaceutical companies with useful information about severe adverse effects. The NHIRD in Taiwan provides the opportunity for outcomes and heath service research. Propensity score analysis simulated the randomization process and tried to eliminate the selection bias for observable factors, and revealed an approximately two-fold increased risk of infection in patients receiving cetuximab. However, functional status and unmeasured factors were not adjusted in propensity score analysis and the association between the cetuximab and infection rate may be overestimated. Instrumental variable analyses could decrease or eliminate the measured and unmeasured biases, and they showed that no statistically significant differences existed between the rate of infections and the average treatment effect of cetuximab.

This study has several limitations. First, the diagnoses of head and neck cancer, infection events, and any other co-morbid conditions are completely dependent on ICD codes. Nonetheless, the National Health Insurance Bureau of Taiwan randomly reviews the charts and interviews patients in order to verify the accuracy of diagnosis. The head and neck cancer patients are further verified by the registry for catastrophic illness patient database. Second, radiotherapy dose and type, cancer stage, and the severity of the infection events cannot be precisely extracted from the NHIRD, which prevented further sub-group analysis. Instrumental variable analysis could eliminate the selection biases from the unmeasured factors. However, it is possible that instrumental variables do not adequately control for unknown confounding factors. Third, chemotherapy that was not approved by the NHI before 2010 but was self-paid by patients, such as taxol (approved on Jan 1^st^, 2011 by the NHI in Taiwan) cannot be extracted from the dataset. Further research studies linking primary hospitalization or ambulatory settings information, such as infection severity, with detailed risk factors, are worth performing in the future.

This study shows that during a one-year follow-up period, cetuximab was not statistically associated with an increased risk of infection by using an instrumental variable analysis. However, older HNC patients using cetuximab may incur an up to 33% infection rate during one year. Therefore, particular attention should focus on older head and neck cancer patients treated with cetuximab.
